# Genetic overlap between idiopathic scoliosis and schizophrenia in the general population

**DOI:** 10.1007/s43390-024-00979-9

**Published:** 2024-10-15

**Authors:** Steven de Reuver, Worrawat Engchuan, Nickie Safarian, Mehdi Zarrei, Jacob A. S. Vorstman, René M. Castelein, Elemi J. Breetvelt

**Affiliations:** 1 Department of Orthopedic Surgery, University Medical Center Utrecht, P.O. Box 85500, 3508 GA Utrecht, The Netherlands; 2 The Centre for Applied Genomics, The Hospital for Sick Children, Toronto, ON Canada; 3 Genetics and Genome Biology Program, Research Institute, The Hospital for Sick Children, Toronto, ON Canada; 4 Department of Psychiatry, The Hospital for Sick Children, 555 University Avenue, Toronto, ON M5G 1X8 Canada

**Keywords:** Idiopathic scoliosis, Schizophrenia, 22q11 deletion syndrome, Pleiotropy, GWAS

## Abstract

**Introduction:**

Adolescent idiopathic scoliosis (AIS) and schizophrenia (SCZ) are two distinct conditions with poorly understood aetiologies that both emerge in otherwise healthy young adolescents. One rare genetic condition associated with both phenotypic outcomes is the 22q11.2 deletion (22q11DS). This microdeletion, encompassing 47 genes, occurs in approximately 1 in 2,148 live births and confers a 20-fold higher risk for both AIS and schizophrenia compared to the general population. In the general population (non-22q11DS carriers), AIS and SCZ have also been reported to be related and genetic studies suggest the involvement of genetic variants implicated in the central nervous functioning. In this study, our objective was to further investigate genetic overlaps between these conditions in the general population. Specifically, we aimed to explore the role of genes within the 22q11.2 region, not only in terms of common variants but also their potential impact on gene networks and biopathways.

**Methods:**

We used summary statistics from three genome-wide association studies (GWAS): two focused on AIS (*n* = 11,210), and one on schizophrenia (*n* = 36,989). To explore potential overlaps between the two conditions, we conducted a comparative analysis on the significance-based ranked single nucleotide polymorphisms (SNPs) that are associated with both AIS and SCZ. Next, we employed in silico analyses to assess gene-networks enrichment for the most significant SNPs and investigate the contribution of genes within the 22q11.2 region. Post-hoc analysis was conducted to explore the biological pathways correlated with SNPs significantly associated with both AIS and SCZ.

**Results:**

The in silico analyses revealed a significant (adjusted-*p* < 0.05) genetic overlap between SCZ and both AIS cohorts. The top 3% of the most significant SNPs associated with both conditions exhibited a distinct enrichment cluster which is unlikely to be a result of chance (*p* < 3e-04). The gene-networks analyses showed a significant overlap of 26–41% with the ones involving genes in the 22q11DS region. However, there was no overlap between SNPs in this region and the most significant SNPs identified in the GWAS.

**Conclusion:**

This study revealed compelling evidence that beyond the shared association with 22q11DS as a rare genetic variant, AIS and SCZ exhibit common genetic risk variants and an overlap of important genes. The gene networks enriched by the most significant SNPs for both conditions also intersect with the ones involving genes in the 22q11DS region. However, SNPs within this region were not overrepresented among the most significant SNPs from GWAS for both conditions. Notably, gene networks linked to the risk for both conditions suggest an involvement of biopathways related to cellular signaling and neuronal development.

**Supplementary Information:**

The online version contains supplementary material available at 10.1007/s43390-024-00979-9.

## Introduction

Idiopathic scoliosis, a prevalent spinal condition, and schizophrenia, a severe mental health disorder, both affect otherwise healthy young adolescents and can significantly impact their quality of life [1, 2]. Despite their co-occurrence in the general population, there is a scarcity of research investigating potential shared risk factors or biological pathways between adolescence idiopathic scoliosis (AIS) and schizophrenia (SCZ). Both conditions are believed to have multifactorial etiology, involving a complex genomic architecture with a wide range of genetic variations, ranging from extremely rare to common variants, each with varying effect sizes [1, 3].

Interestingly, the two conditions have a shared genetic risk variant known as the 22q11.2 deletion syndrome (22q11.2DS) [4]. Despite being classified as a rare genetic disorder, 22q11DS is among the most prevalent of rare recurrent pathogenic copy-number variants (CNVs), with an incidence of 1 in 992 unselected pregnancies and 1 in 2,148 live births [5–7]. Phenotypic manifestations of 22q11.2DS are highly variable and can impact multiple organ systems [5]. Notably, the syndrome is consistently linked to a substantially elevated risk for AIS, with approximately 50% prevalence compared to ~ 3% in the general population, as well as a similarly increased risk for SCZ at ~ 25%, compared to ~ 1% in the general population. This unique association makes 22q11.2DS one of the most significant single genetic risk factors for both conditions [1, 2, 8, 9].

Previous studies have provided evidence that clinical manifestations of 22q11.2DS are truly pleiotropic[10]. The implication is that having one condition associated with 22q11DS does not necessarily increase the risk of developing another condition associated with the same deletion [11, 12] For instance, the early childhood autistic features in children with 22q11.2 deletion was reported not to be linked to an increased risk of subsequent development of psychotic disorders in adults with 22q11DS. Additionally, there are no reports suggesting that AIS and SCZ cluster together within clinical 22q11.2DS cohorts. However, it is worth noting that in a large Swedish general population study, a modest association between the two conditions was found [11]. The association could, in part, be attributed to undetected 22q11.2DS carriers, as scoliosis in 22q11DS carriers is classified differently in the ICD-10. Also, there may be carriers of other (ultra) rare CNVs associated with both conditions, as reported by Mulle et al. (2016) [13]. Nonetheless, considering the rarity of these CNVs, it is improbable that they fully account for the observed association between AIS and SCZ. Thus, it is possible that the two conditions share other (genetic) risk factors, contributing to their association.

While the Swedish population study remains the only investigation into the association between AIS and SCZ to our knowledge, other studies provide evidence of the involvement of the central nervous system (CNS) in the etiology of AIS. These studies have explored abnormal regional cerebral cortical thickness, different relative brain structure volumes and shapes [14–16], as well as CNS functioning, from neurophysiology to proprioception and vestibular functioning [17–19]. Moreover, genetic studies focusing on AIS have indicated the involvement of axonal guidance, CNS development, and neuro-osseous growth modulators in the pathophysiology of the condition [20–22]. Notably, some of the genes implicated by genome-wide association studies (GWAS) in AIS are also associated with neurodevelopmental disorders.

The association between AIS and SCZ in the general population, as well as their co-occurrence in the 22q11DS, could potentially be influenced by CNS functioning and CNS-related genes. To gain a deeper understanding of their genomic architecture and shed light on the phenotypic impact of the 22q11.2 region, identifying additional genetic overlaps between AIS and SCZ is essential. Exploring common genetic variants would serve as a crucial initial step in this direction and may help advance our understanding of disease etiologies.

Here, we examined the potential overlap between gene networks that are strongly associated with both conditions and those involving genes in the 22q11.2 region. Moreover, we sought to explore the biopathways that might play a role in mitigating the shared risk for both conditions based on our findings. It is important to note that this study is exploratory in nature, primarily aimed at generating hypotheses for future investigation.

## Methods

Classical genetic methods like linkage disequilibrium score regression (LDSC) and genomic restricted maximum likelihood (GREML) have limitations in determining the overlap between two conditions if there is a relatively modest sample size and mixed ancestry issues, as is the case in our AIS cohorts. Here, we have applied a novel approach to examine the genetic overlap between AIS and SCZ using summary statistics from GWAS studies.

**Fig. 1:**
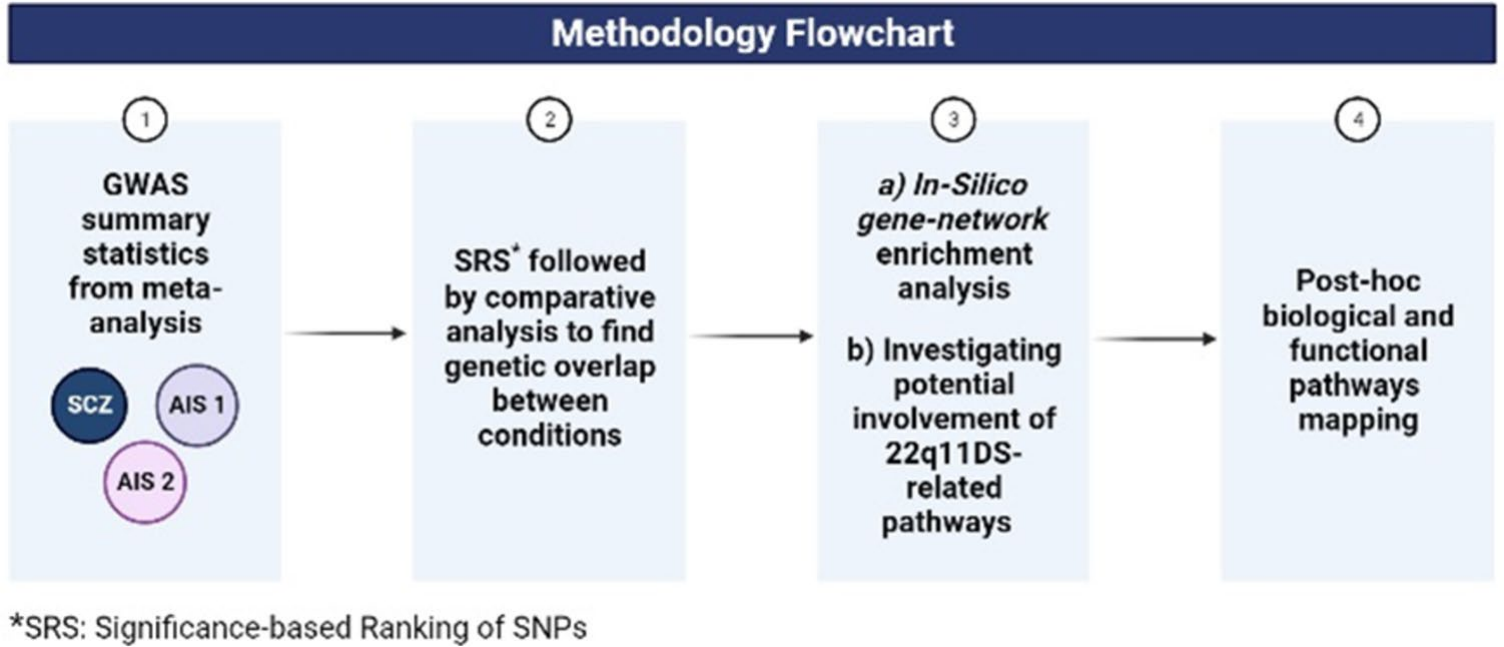
Methodology flowchart

First, we conducted a significance-based ranking of all SNPs, including intergenic, intronic, and exonic SNPs, using the -log10 of p-values. Next, we performed in silico analyses to assess the overlap of gene networks involving the most significant SNPs identified in both GWAS, and those related to genes in the 22q11.2 region. Then, we explored the biological pathways that are associated with the SNPs showing significant association with both conditions. See Fig. 1 for a study overview.

### Study populations

In this study, we used the GWAS summary datasets of AIS and SCZ. The GWAS meta-analysis summary statistics for AIS were downloaded from the GWAS catalog database (https://www.ebi.ac.uk/gwas/downloads/summary-statistics). For the discovery dataset, we used the AIS GWAS summary result published by Khanshour et al. in 2018, (referred to as AIS cohort 1). This dataset includes 7,956 cases and 88,459 controls (https://www.ebi.ac.uk/gwas/publications/30395268) [23]. For the replication dataset (referred to as AIS cohort 2), we obtained another AIS GWAS summary statistics published by Kou et al., in 2019, consisting of 3,254 cases and 63,252 controls (https://www.ebi.ac.uk/gwas/publications/31417091)) [24]. GWAS summary data for SCZ was obtained from the Psychiatric Genomics Consortium (PGC; https://pgc.unc.edu/for-researchers/download-results/). This data was generated by the Schizophrenia Working Group of the PGC based on European and Asian participants for SCZ (36,989 cases and 113,075 controls). Summary demographics of all case–control studies can be found in Supplementary Table 1. The detailed sample information and genotyping, processing, and analyzing procedures for each GWAS data were described in the original publications.

### Step1: SNP overlap between AIS and SCZ

In this step, we used a hypothesis-free approach to compare all SNPs, aiming to identify SNP overlap that could not be explained by random variation, regardless of the statistical tests used. First, we selected SNPs that were present in both AIS 1 and the SCZ cohorts, resulting in 3.35 million SNPs. Subsequently, we ranked the SNPs based on their significance in both cohorts independently, with the least significant SNPs assigned the lowest rank number and the most significant SNPs receiving the highest rank number. Next, we divided each set of SNPs into 30 evenly spaced categories for each condition, ensuring an equal number of SNPs in each subset. We then created a cross table using these categories, and the null hypothesis of no genetic overlap between the two conditions was represented by a random distribution of categories/cells.

We used the heatmap function from the R-package “stats” to generate a heatmap of the 30 × 30 cross-table, suppressing reordering and creating dendrograms. To assess significance, we computed z-scores representing the deviance of the SNP count from the mean for each cell. We considered z-scores below − 4 and above 4 as thresholds for significance, irrespective of the underlying distribution, adopting a conservative approach. Our estimation (see Supplementary Table 1 for details) suggested that meaningful clustering would likely be observed in the right upper quadrant of the 30 × 30 matrix. To address multiple testing, we applied a Bonferroni correction, assuming a normal distribution of the deviance from the mean SNP count per cell. We repeated the entire procedure for AIS cohort 2, which consisted of 5.90 million SNPs. Finally, we conducted a sensitive analysis involving 20,000 random permutations to estimate the probability of observing the same pattern by chance.

### *Step 2: *In silico* validation and 22q11.2 region analysis*

To validate the results obtained in step 1, we conducted an in silico gene–gene interaction analysis to determine the gene network overlap between AIS and SCZ. This analysis focused on the genes associated with the most significant SNPs identified in the GWAS of both conditions. To ensure consistency and avoid any potential sex biases between the disorders, we included only autosomal variants. Additionally, to minimize the inclusion of false positive variants with a pseudo-protective effect (odds ratio; OR < 1), we retained only those with an OR greater than one for further analysis.

We used Web-based ANNOVAR (wANNOVAR; https://wannovar.wglab.org/) [25] to annotate the variants, focusing on those within the upstream and downstream regions of the genes excluding intronic variants. We selected the top 100 genes from each GWAS, using one list as a reference set and the other as a query set of genes. Interaction scores were assigned to genes not present in the reference set using GeneMania (http://www.genemania.org), while for the genes within the reference set, the maximum interaction score was assigned [26]. Subsequently, we used GeneMania to expand the query set, ranging from 100 to 1000 genes. To perform enrichment analysis, we used GSEAPreranked with genes ranked by interaction scores as the pre-ranked list and the expanded query set as the gene set [27]. GSEAPreranked automatically performed multiple test corrections across various expanded query sets.

We also investigated whether the potential overlap in gene networks between AIS and SCZ is influenced by the overlap with genes in the 22q11.2 region. To achieve this, we conducted gene-network analyses between the genes identified as top results from GWAS and the 47 genes located within the genomic range affected by the 22q11DS.

### Step 3: Post-hoc analyses: Biological and functional pathway analysis

Next, we delved into the biological pathways enriched with the SNPs found in cells showing the most significant overlap between SCZ and AIS (see step 1). By exploring these pathways, we aimed to gain insights into how the shared genetic variants may influence the vulnerability to both conditions at the functional level. For pathway enrichment analysis, we used the online portal g:Profiler (https://biit.cs.ut.ee/gprofiler/gost). As input, we used the SNP IDs without any prioritizing within the strata. A conservative adjusted p-value of 0.00005 was considered as the significance threshold. Detailed information about the selected databases for comparison can be found in Supplementary Table 1.

To visualize the results, we utilized Cytoscape (v3.9.1) along with its plugin, EnrichmentMap (v3.3.4), to create a map of enriched biological pathways [28]. In this map, nodes represent biological pathway, and edges depict relationships between them. The relationships were represented using a Jaccard’s index, which quantifies the overlap of genes between the pathways. Only edges with a Jaccard’s index of at least 0.35 were displayed to highlight significant associations. To reduce the density of highly correlated networks, we implemented a step-down approach to make the network less dense and more informative. We ranked the results by their p-values from most to least significant. Subsequently, as we traversed the list, we discarded similar biological pathways with a Jaccard’s index greater than 0.5, retaining the one with the lower p-value in the network. This approach allowed us to integrate and compare the results from both AIS cohorts on the same networks.

Additionally, we compared the biological pathways related to gene networks identified by GWAS with those connected to the genes in 22q11.2 critical region. To achieve this, we extracted a gene subset for each of the gene lists (i.e., top GWAS gene list, and the 47 genes in the 22q11.2 region) based on a GeneMania score above the mean of all GeneMania scores. This subset comprised the genes driving enrichment in the GSEAPreranked analysis. We defined the overlap between the two conditions and between each individual condition and the 22q11.2 gene network. Next, we employed g:profiler to obtain a list of biologically significant pathways (i.e., *p*-value < 0.00005) for each pair of overlaps, such as SCZ and AIS cohort 1 overlap versus SCZ and 22q11.2DS overlap. To assess the level of overlap, we calculated Jaccard’s index for all combinations of gene lists from the GWAS and the gene network from the 22q11.2 region. Finally, we examined the distribution of SNP types in the most significant cells and strata to gain further insights into the genetic variations associated with the identified overlaps.

## Results

### 1 SNP overlap between AIS and SCZ

**Fig. 2:**
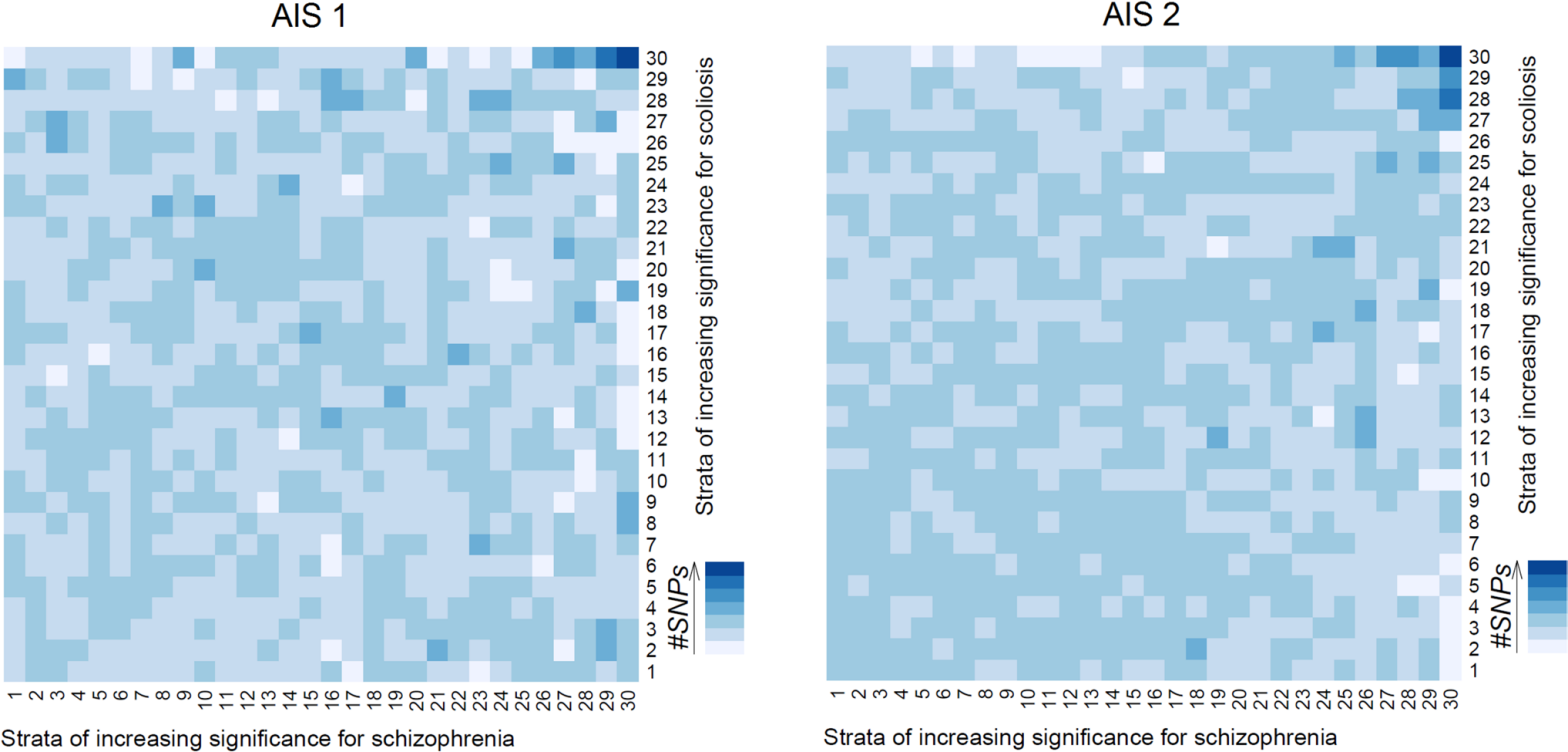
Heatmaps showing the magnitude of SNP overlap between AIS and SCZ. Each cell on the heatmaps represents one of the 30 genomic ranges created to evenly distribute SNPs into categories/groups. Since SNPs were ranked by significance, the first category (i.e., the first cell) on both X and Y axes represent the least significant SNPs, while the 30th cells contain the most significant SNPs. SNPs related to SCZ and AIS are presented on X and Y axes, respectively. The scaled counts of overlapping SNPs (i.e., zscores) are depicted by the gradient of blue colors, where darker blue indicates higher zscores (i.e., more SNPs overlap between conditions**)**

By cross comparing the scaled SNP counts per cell between conditions (Fig. 2), we discovered that the majority of cells display counts close to the mean count. However, there are notable cells containing the most significant SNPs for both conditions, observed in the extreme upper-right quadrant. This pattern was consistent for both AIS1 and AIS2. In the comparison between AIS1 and SCZ, two cells stood out with z-scores exceeding 4, while in the comparison between AIS2 and SCZ, four cells displayed z-scores above 4.

Histograms of the corresponding z-scores for the deviance from the mean cell counts showed a normal distribution of the deviation from the mean count. Notably, all six cells with counts above the threshold displayed z-scores above 5, further affirming the significance of these observations. Next, we applied Bonferroni correction to the p-values for each cell. The corrected p-values for the cells showing the most overlap were all below 3e-04, reinforcing the robustness of our findings. Furthermore, the sensitivity analysis showed that the likelihood of our observations being explained by random chance is exceedingly low, with a probability of 1 in 2 million.

### 2 in silico validation and 22q11.2 region analysis

**Fig. 3:**
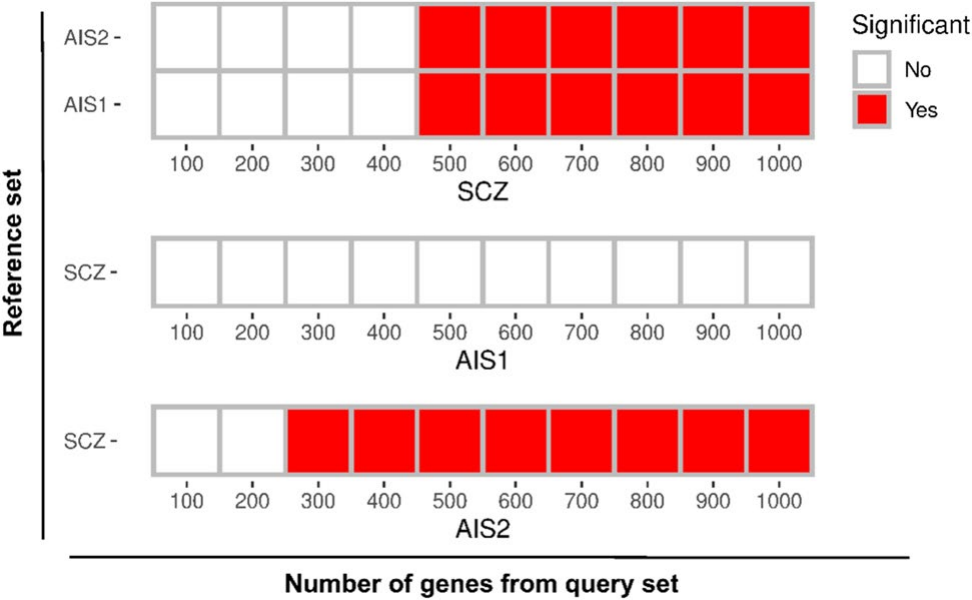
Gene network overlap between conditions. The reference dataset is presented on the Y-axis while the query sets are on the X-axis. Each cubic cell on the x-axis represents a sequential increase of 100 genes in the query set. The magnitude of overlap between the top 100 genes from the reference dataset with the GeneMania-expanded list of genes from the query set is presented as a heatmap. The red color indicates the significance (*p* < 0.05) of overlap

We conducted a gene–gene interaction analysis using the GeneMANIA database, to determine the in-depth interactions of top GWAS hits for each condition. As shown in Fig. 3, the results of GeneMANIA revealed a significant gene network overlap between the two conditions.

When the SCZ cohort served as the reference, we observed a substantial gene network overlap with AIS 2 cohort (used as the query set). The overlap became significant (adjusted *p* < 0.05) when expanding the list of genes in the query set to 300 and beyond (Fig. 3, bottom heatmap). In contrast, increasing the number of genes in AIS cohort 1 did not yield a significant overlap (Fig. 3, middle heatmap). This discrepancy in findings between the two AIS cohorts may be attributed to the fact that compared to AIS1, the AIS 2 cohort processed a higher number of genetic variants (9,059,064 vs. 3,493,832 SNPs) and explained greater variance in the phenotype (4.6% in AIS2 vs. 2.6–2.9% in AIS1). Expanding the gene list from top loci in AIS1 would acquire higher false positives (i.e., acquiring more non-associated genes) than AIS2. Using AIS cohorts as the reference, extending the list of genes to at least 500 and beyond resulted in a significant (p.adjusted *p* < 0.05) gene network overlap between conditions. These findings indicate a pronounced shared functional annotation overlap between the top GWAS hits of AIS and SCZ, which aligns with the findings in part 1.

Interestingly, in the next tier of gene network overlap analysis that focused on the genes in the 22q11.2 region, a substantial overlap was observed between the 22q11.2-related networks and AIS cohort 2, as well as with the SCZ cohort. However, in the case of AIS cohort 1, the overlap only attained statistical significance when the gene list was expanded to include 600 genes. Detailed results from the *In-silico* analysis can be found in Supplementary Table 2 and 3.

### 3 post-hoc analyses: Biological and functional pathway analysis

**Fig. 4:**
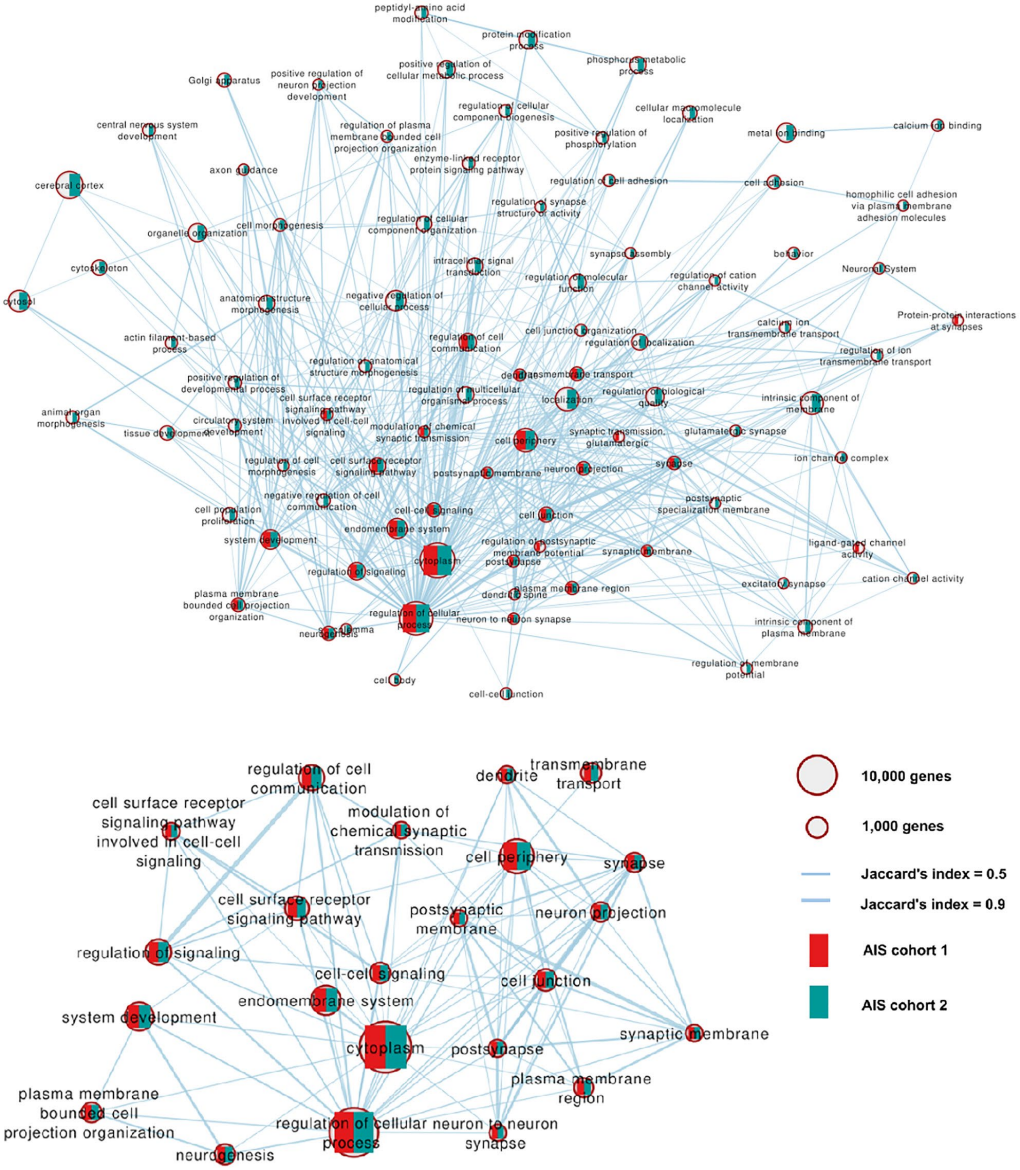
Biopathways overlap analysis. The biological pathways enriched by overlapping genetic variants between SCZ and both AIS cohorts are presented. The nodes represent the biopathways and the connecting lines indicate different correlations. Functional associations were investigated using GeneMania. The red and green traced depict the AIS 1 and AIS 2 cohorts, respectively. **A** the significant pathways for either AIS cohort, **B** A zoom-in view of the significant pathways for both AIS cohorts. Significance: *p* < 0.05

The biopathway analysis between the top SNPs of AIS and SCZ revealed multiple significant overlapping biological pathways (Fig. 4). Notably, the most significant pathways are closely related and indicate the involvement of early brain development mechanisms, as well as cellular and neuron signaling. When comparing the results for the AIS 1 and AIS 2 cohorts, we observed several different biological pathways. However, it is noteworthy that the center of the closely related cluster of biological pathways is shared between the two analyses. It is intriguing to note that most of the identified pathways are primarily associated with the brain, and no pathways related to immunology were detected.

In Supplementary Table 2 and 3, we present the proportions of SNP types, indicating that, similar to all GWAS, a substantial proportion of the SNPs in the cells identified in part 1 are located in intergenic and non-coding regions. Upon investigating the biological pathways of gene-network overlaps between AIS cohort 1 to SCZ, as well as with genes in 22q11.2 region, we discovered that the two overlaps exhibited a 40% shared similarity (Jaccard’s index) in biological pathways. Notably, these shared pathways were related to synapse, neuron projection, and cell junctions. In contrast, the overlaps between AIS cohort 1 and SCZ showed a more specific association with system development and cell signaling.

## Discussion and conclusion

In this study, we investigated the potential genetic overlap between AIS and SCZ in the general population, focusing on common genetic variants. Through analysis of GWAS summary statistic data, we uncovered a significant overlap between the SNPs associated with AIS and SCZ. This overlap suggests that a portion of the common genetic variants associated with AIS also play a role in SCZ. In silico analyses also confirmed that genes important in AIS and SCZ exhibited a greater degree of overlap than what would be expected by chance across the entire genome. Moreover, only a small number of SNPs in the 22q11.21 region are present in cells/categories containing the most significant SNPs for both AIS and SCZ (0.017%). This suggests that the observed overlap in gene networks is unlikely to be driven by common variants in the 22q11.21 region. However, it is interesting to observe that the enriched gene networks for both AIS and SCZ do overlap with gene networks enriched for genes in the 22q11.21 region. Lastly, functional analyses revealed that common variants associated with both AIS and SCZ, are highly enriched in regulatory and synaptic/neuron function pathways.

In addition to the theories discussed in the introduction regarding the potential involvement of the central nervous system (CNS) in the etiology of AIS, several other concepts and theories have been postulated in the literature. For instance, associations have been observed between AIS and conditions such as low BMI, low leptin levels, and osteopenia, but it remains unclear whether these occurrences are caused or affected by AIS [20, 21, 29, 30]. The challenge lies in the fact that most studies are conducted on patients or models with already established scoliosis, making it difficult to infer causality. From a biomechanics perspective, axial rotational instability [31], may also play a role in AIS. This instability arises when posteriorly directed shear loads are applied to the spine [32], which are specific to the unique upright spines of humans [33]. Interestingly, AIS is exclusively observed in humans [34]. Recently, a prospective study showed that the relative size and angulation of the posteriorly directed spinal segments contribute to an increased risk of scoliosis. Moreover, other biomechanical concepts have been proposed to explain scoliosis risk. One concept suggests a mismatch between the growth of the vertebral column height and either the spinal cord or the surrounding muscles and tendons. This mismatch may act as a tether, leading to the buckling of the spine [35–37]. Another theory revolves around asymmetrical loading, resulting in an unclear yet significant Hueter–Volkmann effect, leading to asymmetric bone growth [38, 39]. Metabolic theories propose the involvement of platelet calmodulin dysfunction [40] and melatonin pathway dysfunction [29, 41]. Additionally, central cord tethering leading to lower cerebral tonsils has also been implicated as a potential factor in the development of AIS.

In our study, we found that the pathways significantly enriched in two distinct AIS cohorts predominantly involve cell (membrane) processes and signaling. However, these pathways also encompassed multiple synaptic and other neuron functioning and development, suggesting the involvement of fundamental cell and neuro-regulatory mechanisms in the etiology of AIS.

The primary limitation of this study is the relatively small sample size of available GWAS data for AIS, especially for AIS1. As we used summary statistics, we were unable to combine the two AIS GWAS datasets, potentially impacting our ability to detect all relevant genetic associations. This might explain the need to expand the number of genes to 600 for the in silico analyses to detect significant overlap between 22q11.21 genes for AIS cohort 1. Additionally, this could also shed light on why the comparison between SCZ and AIS1 resulted in a smaller effect size and trend-level p-values. Despite these limitations, we are confident in the main finding of this study, which is supported by the exceptionally low likelihood of obtaining similar results through random sampling (Supplementary data 1). It’s worth noting that despite the mixed ancestral background in the AIS cohorts, we observed a strong consistency in the results for both AIS cohorts 1 and 2.

In conclusion, this study represents the first report of genetic overlap between AIS and SCZ in the general population., with a specific focus on common genetic variants. Our findings align with previous observations of shared rare genetic risk between these conditions while providing additional evidence for the presence of shared common risk alleles.

This exploratory study generates novel hypotheses concerning the etiopathogenesis of AIS and highlights the potential shared genetic risk and genomic architecture of both AIS and SCZ. It suggests that subtle alterations in neuron and brain development may contribute to the risk for AIS, among various multifactorial causes. Furthermore, besides 22q11DS, a group of common genetic variants appears to be associated with an increased risk for both AIS and SCZ. This suggests, the existence of a shared genetic risk that encompasses the entire spectrum of genetic variation, ranging from rare to common variants. An interesting question arises: does this shared genetic risk exert its influence through common biological mechanisms shared by both conditions, or does it operate more indirectly by modifying regulatory elements in the genome?

Another intriguing finding is that while SNPs in the 22q11.21 region do not fully account for the observed genetic overlap at the level of common genetic variants, certain gene networks enriched with genes carrying this shared genetic risk for AIS and SCZ also display an enrichment of genes located in the 22q11.21 region. This suggests that within these gene networks, some genes may be influenced by variations in common genetic variants, which could have phenotypic consequences. On the other hand, for other genes within the same network, changes in gene dosage may have functional effects, similar to what is observed for some genes in the 22q11.2 region.

Whether the non-coding SNPs, which are linked to an increased risk for both conditions, contribute to a broader functional (regulatory) genetic network represents an exciting novel hypothesis that requires further exploration. Moreover, the biological pathways enriched by SNPs associated with both AIS and SCZ are pathways that have been well-conserved throughout evolution. This raises questions about why and how these pathways are involved in the shared risk for two uniquely human conditions. Unraveling the reasons and mechanisms behind this phenomenon may offer valuable insights into the complex nature of these disorders and provide a deeper understanding of their shared genetic basis. In addition, the potential clinical importance of these findings lies in the possibility of using genetic information to identify individuals at increased risk for conditions such as AIS and SCZ which would open opportunities for early interventions and mitigation of long-term outcomes. In this respect, the key challenge is answering the question of why predictive properties of genetic risk models for both AIS and schizophrenia are much lower than expected based on the strong hereditary component found in family and twin studies. The current challenges with achieving clinically useful genetic risk prediction metrics do not imply that they will never be useful in clinical practice. It simply suggests that more fundamental research is required. The complexity of the genomic architecture might have been underestimated, and other approaches are likely needed. For schizophrenia, many initiatives are ongoing to elucidate this complexity, like focusing on the impact of a regional excess of low-frequency variants[42]. The present study, by establishing genetic overlap between AIS and schizophrenia, and the possible shared biological pathways, contributes to a better understanding of the complex architecture of both conditions.

## Supplementary information

Below is the link to the electronic supplementary material.

Supplementary file1 (DOCX 16 KB)

Supplementary file2 (DOCX 14 KB)

Supplementary file3 (DOCX 14 KB)

## Data Availability

Data was publically available, statistical pipelines can be requested via contacting the coresponding authors.
