# Supplementary material for: Genetic overlap between idiopathic scoliosis and schizophrenia in the general population
Source: Spine Deform. 2024 Oct 15;13(2):413–22. doi: 10.1007/s43390-024-00979-9 (PMC11893639; doi:10.1007/s43390-024-00979-9)
Supplement: Supplementary file 1 — Supplementary file1 (DOCX 16 KB) [file 43390_2024_979_MOESM1_ESM.docx]

**Supplementary Table 1**

| ***Stage*** | **Disorder** | **cohorts** | **subjects** | **cases** | **controls** | **# of SNPs** | **Genotyping Platform** | **Reference** |
| --- | --- | --- | --- | --- | --- | --- | --- | --- |
| ***Discovery*** | AIS | JP1 | 12840 | 2104 | 10736 | 5,871,515 | Illumina Human (Hap550, Hap600,OmniExpress) Array | [PMID: 30395268](https://pubmed.ncbi.nlm.nih.gov/30395268) |
|  |  | HK | 539 | 191 | 348 | 4,834,299 | Affymetrix 6.0 Array and 500k Array |  |
|  |  | Texas-GWAS1 | 2802 | 546 | 2256 | 5,964,873 | Illumina Human (CNV370,Hap300,Hap550) Array |  |
|  |  | Texas-GWAS2 | 1146 | 441 | 705 | 6,116,547 | Illumina HumanOmniExpress Array |  |
|  |  | Texas-GWAS3 | 8358 | 406 | 7952 | 6,026,942 | Illumina HumanCoreExome Array |  |
|  |  | MO1 | 7791 | 110 | 7681 | 6,069,468 | Affymetrix 6.0 Array |  |
|  |  | JP2 | 59552 | 2737 | 56815 | ? | Invader assay, Illumina Human (OmniExpressExome, OmniExpress, Exome) Array |  |
|  |  | Swedish-Danish | 3387 | 1421 | 1966 | ? | MassARRAY® System combined with iPLEX® chemistry, Infinium® Global Screening Array and Illumina Human Omni Express Exome chip. |  |
|  | SCZ | European | 74626 | 32405 | 42221 | ? | ? | [PMID: 25056061](https://pubmed.ncbi.nlm.nih.gov/25056061) |
|  |  | Trios | 2470 | 1235 | 1235 | ? | ? |  |
|  |  | Asian | 48675 | 1836 | 46839 | ? | ? |  |
| ***Replication*** | AIS | GWAS1 | 4233 | 1254 | 2979 | 9,272,622 |  | [PMID: 31417091](https://pubmed.ncbi.nlm.nih.gov/31417091) |
|  |  | GWAS2 | 8472 | 819 | 7653 | 9,312,384 |  |  |
|  |  | GWAS3 | 66506 | 3254 | 63252 | 9,295,288 |  |  |
| ***Total*** |  | | 301397 | 48759 | 252638 |  |  |  |
