# Supplementary material for: Genetic overlap between idiopathic scoliosis and schizophrenia in the general population
Source: Spine Deform. 2024 Oct 15;13(2):413–22. doi: 10.1007/s43390-024-00979-9 (PMC11893639; doi:10.1007/s43390-024-00979-9)
Supplement: Supplementary file 2 — Supplementary file2 (DOCX 14 KB) [file 43390_2024_979_MOESM2_ESM.docx]

**Supplementary Table 2. Enriched gene-gene interaction networks overlap between SCZ and AIS.**

| **Extension** | **Comparison (Reference – Query)** | | | | | | | |
| --- | --- | --- | --- | --- | --- | --- | --- | --- |
|  | **AIS1 – SCZ** | | **SCZ – AIS1** | | **AIS2 – SCZ** | | **SCZ - AIS2** | |
|  | **NES** | **P.adj** | **NES** | **P.** | **NES** | **P** | **NES** | **P** |
| 100 | 0.86 | 0.9 | 0.91 | 0.7 | 1.0 | 0.5 | 1.1 | 0.2 |
| 200 | 0.93 | 0.8 | 1.02 | 0.4 | 1.03 | 0.3 | 1.14 | 0.1 |
| 300 | 1.03 | 0.3 | 1.03 | 0.3 | 1.09 | 0.1 | 1.19 | **0.03** |
| 400 | 1.07 | 0.1 | 1.05 | 0.3 | 1.08 | 0.1 | 1.18 | **0.01** |
| 500 | 1.14 | **0.005** | 1.08 | 0.2 | 1.12 | **0.01** | 1.16 | **0.03** |
| 600 | 1.15 | **0.001** | 1.08 | 0.1 | 1.17 | **> 0.00001** | 1.15 | **0.02** |
| 700 | 1.16 | **> 0.00001** | 1.09 | 0.09 | 1.18 | **> 0.00001** | 1.15 | **0.01** |
| 800 | 1.19 | **> 0.00001** | 1.07 | 0.1 | 1.19 | **> 0.00001** | 1.16 | **0.005** |
| 900 | 1.19 | **> 0.00001** | 1.07 | 0.1 | 1.22 | **> 0.00001** | 1.14 | **0.008** |
| 1000 | 1.19 | **> 0.00001** | 1.06 | 0.1 | 1.22 | **> 0.00001** | 1.15 | **0.005** |

*Abbreviation: NES = Normalized Enrichment Score, P.adj = Adjusted p-value
** bold text indicates significant result.
