# Supplementary material for: Genetic overlap between idiopathic scoliosis and schizophrenia in the general population
Source: Spine Deform. 2024 Oct 15;13(2):413–22. doi: 10.1007/s43390-024-00979-9 (PMC11893639; doi:10.1007/s43390-024-00979-9)
Supplement: Supplementary file 3 — Supplementary file3 (DOCX 14 KB) [file 43390_2024_979_MOESM3_ESM.docx]

**Supplementary Table 3. The gene networks involving genes located in the 22q11.2 region are also shared with both SCA and AIS.**

| **Extension** | **Query** | | | | | |
| --- | --- | --- | --- | --- | --- | --- |
|  | **SCZ** | | **AIS1** | | **AIS2** | |
|  | **NES** | **P** | **NES** | **P** | **NES** | **P** |
| 100 | 1.01 | 0.4 | 1.04 | 0.2 | 1.10 | **0.05** |
| 200 | 1.09 | **0.04** | 1.02 | 0.3 | 1.18 | **> 0.00001** |
| 300 | 1.12 | **0.005** | 1.06 | 0.09 | 1.22 | **> 0.00001** |
| 400 | 1.10 | **0.01** | 1.04 | 0.2 | 1.23 | **> 0.00001** |
| 500 | 1.11 | **0.001** | 1.05 | 0.1 | 1.23 | **> 0.00001** |
| 600 | 1.13 | **> 0.00001** | 1.06 | **0.04** | 1.23 | **> 0.00001** |
| 700 | 1.16 | **> 0.00001** | 1.04 | 0.08 | 1.24 | **> 0.00001** |
| 800 | 1.17 | **> 0.00001** | 1.04 | 0.09 | 1.25 | **> 0.00001** |
| 900 | 1.18 | **> 0.00001** | 1.04 | 0.07 | 1.27 | **> 0.00001** |
| 1000 | 1.19 | **> 0.00001** | 1.04 | 0.08 | 1.28 | **> 0.00001** |

*Abbreviation: NES = Normalized Enrichment Score, P = Corrected p-value
** bold text indicates significant result
